# Supplementary material for: Neighborhood Deprivation and Racial Disparities in Early Pregnancy Impaired Glucose Tolerance
Source: Int J Environ Res Public Health. 2023 Jun 19;20(12):6175. doi: 10.3390/ijerph20126175 (PMC10298257; doi:10.3390/ijerph20126175)
Supplement: Supplementary file 1 [file ijerph-20-06175-s001.zip › ijerph-2394639-supplementary.pdf]

Table S1. Characteristics of pregnant, non-diabetic patients residing in Philadelphia included in and excluded from the analytic cohort due to missing BMI (n= 1,824) or additionally missing hemoglobin A1c (n= 7,901) data.

	All (n=20,385)	Included in analysis (n=10,642)	Excluded from analysis (n=9,743)	P-value
Characteristics				
Age (years), mean [SD]	30.0 [5.8]	30.2 [5.7]	29.8 [5.9]	<0.001
Obese (≥30 kg/m^2)	5,322 (26.1)	3,384 (31.8)	1,938 (24.5)	<0.001
Race and ethnicity				
Non-Hispanic Black	10,019 (49.1)	5,219 (49.0)	4,800 (49.3)	0.73
Non-Hispanic White	6,222 (30.5)	3,237 (30.4)	2,985 (30.6)	
Another race or ethnicity	4,144 (20.3)	2,186 (20.5)	1,958 (20.1)	
Public Insurance	10,670 (52.3)	5,181 (48.7)	5,489 (56.3)	<0.001
Smoked during pregnancy	787 (4.2)	633 (6.0)*	154 (1.9)^	<0.001
Nulliparous	8,999 (44.1)	4,755 (44.7)	4,244 (43.6)	0.11

Data presented as n (column %) unless otherwise specified.

*missing n= 140

^missing n= 1,557

Table S2. Results of sensitivity analysis including only individuals with pregnancy weight height measured (no imputation of BMI) (n=8,522)

**Models of neighborhood deprivation with IGT**	**OR**	**(95% CI)**
M0 = Unadjusted	1.39	(1.22, 1.59)
M1 = M0 + age, insurance, parity	1.43	(1.31, 1.56)
M2 = M1 + obesity	1.29	(1.20, 1.40)
M3 = M1 + race	1.14	(1.05, 1.23)
M4 = M1 + race + obesity	1.07	(0.99, 1.15)
Black patients		
M0 = Unadjusted	1.08	(0.96, 1.21)
M1 = M0 + age, insurance, parity	1.19	(1.06, 1.33)
M2 = M1 + obesity	1.15	(1.02, 1.29)
White patients		
M0 = Unadjusted	1.14	(0.93, 1.40)
M1 = M0 + age, insurance, parity	1.07	(0.86, 1.33)
M2 = M1 + obesity	0.98	(0.77, 1.23)
**Models of neighborhood deprivation with obesity**		
M0 = Unadjusted	1.76	(1.46, 2.12)
M1 = M0 + age, insurance, parity	1.57	(1.38, 1.79)
M2 = M1 + race	1.39	(1.27, 1.53)
Black patients		
M0 = Unadjusted	1.08	(0.99, 1.18)
M1 = M0 + age, insurance, parity	1.13	(1.03, 1.23)
White patients		
M0 = Unadjusted	1.76	(1.44, 2.15)
M1 = M0 + age, insurance, parity	1.53	(1.26, 1.85)
